# Model-Supported Radiotherapy Personalization: *In silico* Test of Hyper- and Hypo-Fractionation Effects

**DOI:** 10.3389/fphys.2018.01445

**Published:** 2018-10-15

**Authors:** Antonella Belfatto, Barbara Alicja Jereczek-Fossa, Guido Baroni, Pietro Cerveri

**Affiliations:** ^1^Department of Electronics, Information and Bioengineering, Politecnico di Milano, Milan, Italy; ^2^Department of Oncology and Hemato-Oncology, University of Milan, Milan, Italy; ^3^Division of Radiotherapy, European Institute of Oncology, Milan, Italy

**Keywords:** radiotherapy, cervical cancer, hypofractionation, mathematical model, simulation, oxygenation

## Abstract

The need for radiotherapy personalization is now widely recognized, however, it would require considerations not only on the probability of control and survival of the tumor, but also on the possible toxic effects, on the quality of the expected life and the economic efficiency of the treatment. In this paper, we propose a simulation tool that can be integrated into a decision support system that allows selection of the most suitable irradiation regimen. We used a macroscale mathematical model, which includes active and necrotic tumor dynamics and the role of oxygenation to simulate the effects of different hypo-/hyper-fractional regimens using retrospective data of seven virtual patients from as many cervical cancer patients used for its training in a previous study. The results confirmed the heterogeneous response across the patients as a function of treatment regimen and suggested the tumor growth rate as a main factor in the final tumor regression. In addition to the maximum regression, another criterion was suggested to select the most suitable regimen (minimum number of fractions to achieve a regression of 80%) minimizing the toxicity and maximizing the cost-effectiveness ratio. Despite the lack of direct validation, the simulation results are in agreement with the literature findings that suggest the need for hypo-fractionated regimens in case of aggressive tumor phenotypes. Finally, the paper suggests a possible exploitation of the model within a tool to support clinical decisions.

## Introduction

According to the World Health Organization (WHO), cancer is the second leading cause of mortality, and has been responsible for one in six deaths in 2015 worldwide. This is a major burden in both developed and developing countries, with around 14 million new cases in 2012 and an increase of 70% for the next 20 years (Soerjomataram et al., 2012). There are two main research paths available to address this problem. The first is to develop new treatment options by investigating the mechanisms underlying the evolution of the tumor. The second is to optimize and personalize treatments in clinical practice (e.g., drugs, surgery, and irradiation) to address the heterogeneous response of the tumor to the therapy. Among treatment options, radiotherapy is the most applied because it can be used it can be used either to reduce the extent of the tumor before proceeding with surgery (Contin et al., 2014) or to irradiate the resection margins post-surgery (McGale et al., 2014). It can be also used as a palliative therapy (Chow et al., 2007) and as an elective treatment alone or concomitantly with adjuvant chemotherapy (Haddad et al., 2013; Goldstein and Kastan, 2015). External Beam Radiation Therapy (EBRT) is usually delivered to the patients by means of multiple fractions characterized by the nominal dose that must be conveyed to the region of interest including visible tumor and micro-lesions (Burnet et al., 2004). Conventional treatment consists of 1.8–2 Gy fractions delivered 5 days a week, a therapeutic regimen established in early radiobiological studies to maximize the curative effect while reducing toxicity (Colombo et al., 2012).

Recently, the identification of patient-specific genomics and radiomics (omics) biomarkers has suggested the possibility of exploiting altered regimens (Ahmed et al., 2014). An accurate and personalized approach to EBRT planning would require at least two steps: (1) definition of the most suitable fractionation program, including the nominal dose value per fraction, based on patient-specific characteristics; (2) accurate delivery of the nominal dose taking into account the anatomical-pathological changes between fractions and intra-fraction organ movement (Seregni et al., 2012). Regarding dose delivery, irradiation is usually carefully planned by optimizing the beam entry and activation strategies to administer the tumor with the prescribed amount of dose while sparing the organ at risks (OAR). The dose profile can be adjusted according to slow morphological changes (inter-fraction) using a plan-of-the-day approach (Heijkoop et al., 2014; Jones et al., 2015; Sharfo et al., 2016) while faster dynamics (e.g., respiration) can be addressed by means of time resolved images (Keall et al., 2006; Schaerer et al., 2012; Paganelli et al., 2015). In other words, the level of treatment personalization in dose delivery management can be quite impressive. On the contrary, once the staging of the tumor has been defined and radiotherapy or radiochemotherapy is selected accordingly, the amount of irradiation dose to be administered during the fractionated treatment often derives from the general guidelines and the structure protocol (Colombo et al., 2012; Prokopiou et al., 2015).

In the light of this clinical context, mathematical models of tumor evolution and response to treatment could play an important role allowing the customization of radiotherapy simulating different irradiation protocols and thus supporting the selection of the most effective (Stamatakos and Dionysiou, 2009). Mathematical models can range from statistical methods to calculate the tumor control probability (TCP) and normal tissue complication probability (NTCP) (Thames et al., 1983; Lyman, 1985) to multiscale 3D models of cellular and subcellular mechanisms that regulate tumor dynamics (Bellomo et al., 2004; Stamatakos and Dionysiou, 2009; Boondirek et al., 2010). Radiobiological models that evaluate the effects of radiotherapy are routinely used in clinical practice. The best known is the Linear Quadratic (LQ) model, which expresses the surviving fraction of a cell population as a function of the irradiated dose and tumor-/patient- specific radiosensitivity parameters. The LQ model is also the starting point of the biological effective dose (BED) computation, a measure that describes the biological effect of radiation according to the specific treatment schedule (Fowler, 1989, 2014). However, it is known that models failing to incorporate tumor repopulation are not suitable for describing the overall tumor evolution (Stocks et al., 2014). The model complexity increases even further if specific aspects are taken into account (for example cancer metabolism deregulation, genomic factors) (Ghaffari et al., 2015; Oberhardt and Gianchandani, 2015). To properly address the problem of training and validation for a complex model, it is necessary to collect morphological and functional data. Therefore, despite the greater realism and detail of the 3D multiscale models, simpler macroscale models focusing on the scalar evolution of the tumor volume can be considered (Hogea et al., 2008; Huang et al., 2010; Cornelis et al., 2013).

In previous studies, we presented different macroscale models of tumor growth and response to radiotherapy, trained and tested on both animal and clinical data (Belfatto et al., 2015, 2016a,b, 2017). In this work, we aimed at simulating the effects of hyper- and hypo-fractionation schemes together with the conventional schedule using the most complete of the proposed model formulations (Belfatto et al., 2017). The adopted model, including active and necrotic tumor cells and oxygenation dynamics, had already been trained on the volume data of seven patients, diagnosed with cervical cancer, who were treated using the standard 1.8G/28-fraction EBRT regimen and monitored using 3D-Doppler images. The simulated treatments, with the same overall BED, were evaluated in terms of final tumor regression. Further possible criteria for selecting the optimal regimen have been identified.

## Materials and Methods

### Model Definition, Training, and Validation

The equations regulating the evolution of active and necrotic portions were extensively described in our previous work (Huang et al., 2010). Hereafter, we summarize the overall model structure briefly. The tumor volume is considered completely active and growing before the treatment start (*t*_0_), while the irradiation causes the decrease of the active tumor (*V_a_*) and the occurrence of necrosis. The active volume grows spontaneously according to the Gompertz (1825) model featuring two main parameters, the growth rate (ρ) and the carrying capacity (*k*). Since the latter describes the maximum amount of viable tumor sustainable by the tissue, we assumed it to be reduced due the vasculature damage following irradiation [*k*(*t* + 1) = *k*(*t*).*SF_v_*], given that the carrying capacity before treatment is $\hat{k}$. The surviving fraction of the vasculature (*SF_v_*) and the one of the active tumor (*SF_t_*) are assessed by means of the LQ formula (Fowler, 1989):

(1) $$\mathrm{SF}=e^{-\alpha \gamma d\left(1+\frac{d}{\alpha /\beta }\right)}$$

where α (Gy^−1^) is the radiosensitivity and α/β (Gy) is 10 and 3 for the tumor and vasculature, respectively. We considered an additional coefficient γ accounting for the multiple cell killing required to impair the vascular efficiency, therefore γ = 1 for the tumor while γ is positive and lower than 1 for the vasculature. The radiosensitivity depends on the average oxygen partial pressure (*PO*_2_) within the tumor volume as widely recognized in the literature (Deschner and Gray, 1959; Fyles et al., 1998; Karlsson, 2004). We defined a linear relation between the two variables as:

(2) $$\alpha (t)=\alpha_{\min}+\frac{{\mathrm{PO}}_{2}(t)}{100}\cdot (\alpha_{\max}-\alpha_{\min})$$

where α*_min_* = 0.001 Gy^−1^ and α*_max_* = 0.3 Gy^−1^ in order to bound the surviving fraction as 0.5 < *SF_t_* < 1 considering a standard dose per fraction (1.8 Gy) and the selected α/β ratio (10 Gy). The oxygen pressure is determined by the efficiency of the vasculature and the amount of active cells consuming oxygen, therefore we linked *PO*_2_ to both? (index of the environment status) and *V_a_* as follows:

(3) $${\mathrm{PO}}_{2}(t)=\frac{k(t)-V_{a}(t)}{k(t)}100$$

Finally, the necrotic volume was simply reabsorbed, starting from the corresponding irradiation day (*t_i_*), according to an inverse exponential law defined by the half-time of dead cell clearance (*T*_1/2_). The overall discrete equation system, considering a *N*-fraction treatment, is therefore defined as:

(4) $$\left[\left|a(t)+\rho \log_{V_{a}(t+1)}^{V}=\left(\frac{k(t)}{V_{a}(t)}\right)V_{a}(t\right)\right]\cdot {\mathrm{SF}}_{t}$$

(5) $$V_{n}(t+1)=V_{a}(t)(1-{\mathrm{SF}}_{t})+\sum_{i=1}^{N}V_{n,i}e^{-\log(2)\frac{t-t_{i}}{T_{1/2}}}$$

(6) $$V_{t}(t+1)=V_{a}(t+1)+V_{n}(t+1)$$

**FIGURE 1:**
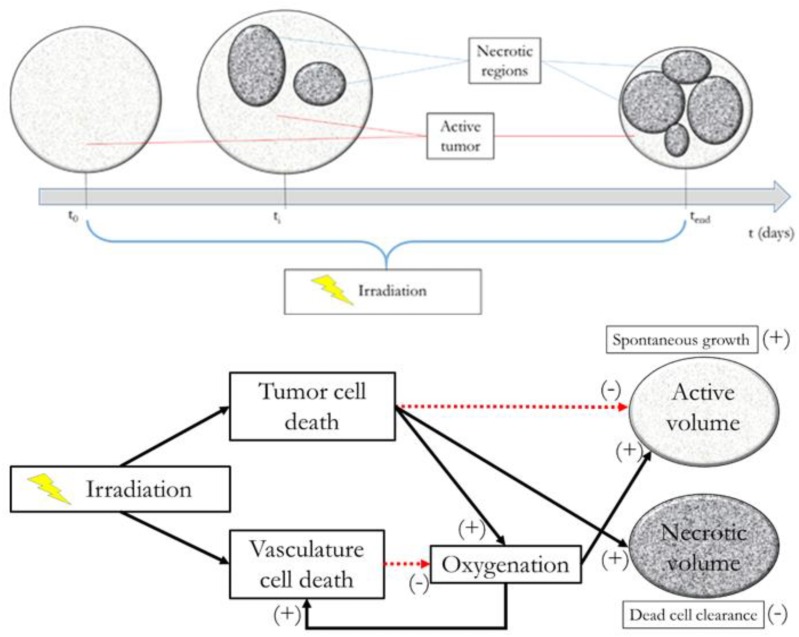
Model at glance. Upper panel: example of tumor evolution through time. Lower panel: active volume, necrotic volume, irradiation, and oxygenation interplay diagram.

The interplay among all the above mentioned variables is graphically described in **Figure 1**, along with an example of possible tumor evolution through time during the treatment administration. The system featured four free parameters, namely ρ, $\hat{k}$, *T*_1/2_, and γ, while all other quantities were set according to literature values as described in the text where they were first introduced. Free parameters underwent patient-specific optimization by means of a custom Montecarlo algorithm implemented in MATLAB^®^ (The MathWorks, Inc., Natick, MA, United States). The growth rate was searched in the interval 0.01 < ρ < 0.2, the initial carrying capacity $\hat{k}$ was allowed to span between 1 and 3 times the initial tumor volume (100 < $\hat{k}$ < 300), while the dead cells clearance time (*T*_1/2_) was bounded between 2 and 30 days (Belfatto et al., 2017). Finally, γ, determining *SF_v_*, was allowed to range in the [0 .. 1] interval. This was set to limit the lethal effects of irradiation to the vasculature according to the rationale of radiotherapy that healthy cells are less sensitive than the tumor ones. The model was optimized according to the methodology described in Belfatto et al. (2017) using the tumor volume evolution (*V_t_*) of seven woman (age range: 41–81 years), listed from A to G, affected by uterine cervical cancer. Details of the estimated parameters are depicted in **Table 1**. 3D-Doppler indexes of vascularization and flow, namely the vascularization index (VI), the Flow Index (FI and the Vascularization-Flow Index (VFI), were exploited to validate the oxygenation dynamics independently. Despite being related to the oxygen availability, the aforementioned indexes do not correspond to the actual value of oxygen pressure in the tumor. Therefore, the correlation values between each of VI, FI, VFI and the model-based oxygenation values (*PO*_2_) were considered as a measure of the model ability to mimic the interplay among volume (active and necrotic) and environmental condition dynamics.

**Table 1:** Estimated model parameters, namely ρ, *T*_1/2_, $\hat{k}$, and, γ computed in the Montecarlo optimization procedure.

Patient	ρ (rate)	*T*_1/2_ (days)	$\hat{k}$ (%)	γ (rate)
A	0.17	14	220	0.9
B	0.07	28	150	0.7
C	0.08	4	125	0.7
D	0.02	22	300	0.9
E	0.02	30	140	0.8
F	0.10	30	120	0.6
G	0.10	30	120	0.7

The patients were scheduled for chemo-radiation exclusive therapy because of histologically proven advanced cervical cancer according to International Federation of Gynecology and Obstetrics (FIGO) staging (1 IIA, 2 IIB, 3 IIB N^+^, and 1 IVA) from September 2014 to July 2015. The study was part of one current research program on image-guided radiotherapy for gynecological cancer reported to and approved by multidisciplinary gynecological oncology board (Institutional Ethics Committee: notification no. 86/11) of the European Institute of Oncology (Milan, Italy). All patients gave written informed consent for the treatment and for the use of the anonymized data for research or educational purpose. The data were collected in European Institute of Oncology institutional database (RTP R036-000-BRACHI-GINE).

### Treatment Simulation Protocol

Theoretically, there are no limits to the treatment schemes that could be simulated by means of the model described. However, some considerations are in order: (a) the amount of dose delivered should suffice to treat the tumor or at least significantly reduce its volume; (b) the healthy tissue toxicity should be avoided; and (c) the model was trained and tested on 1.8 Gy × 28 fractions protocols only. We decided to leave the standard irradiation schedule of 5 days per week unvaried while analyzing the effect of changes in the dose profile.

**FIGURE 2:**
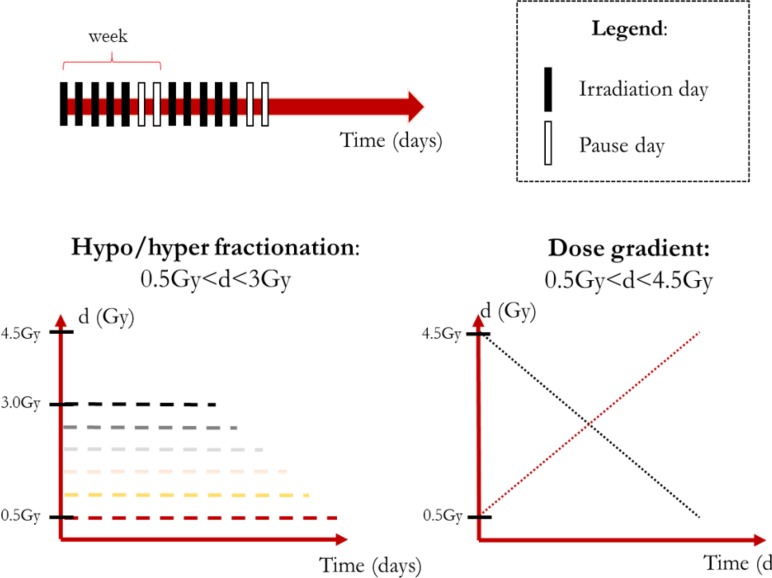
Summary of the simulation scheme.

In order to provide a reasonable amount of irradiation to the patient, we imposed BED ≅ 60 Gy as this is about the BED value for a 1.8 Gy/fraction treatment delivered in 28 sessions considering α/β = 10 Gy. Although setting α/β = 10 Gy is in agreement with both literature and clinical practice (Dale, 1996), the ratio is actually dependent on the tissue type, dose amount and even oxygenation (Williams et al., 1985; Fowler, 2001; Lee et al., 2014). Therefore, the simulations should be performed acknowledging the limits of the model extrapolation abilities. Two main types of treatments were defined: (1) hypo/hyper fractionated schemes featuring a constant value of dose per fraction; (2) non-constant schemes featuring dose gradients (increasing/decreasing dose trends). According to the previous consideration the dose boundaries were defined for the constant dose treatment (0.5 Gy ≤*d*/fraction ≤ 3 Gy) and for the non-constant dose ones (0.5 Gy ≤*d*/fraction ≤ 4.5 Gy, average dose: 2.5 Gy) as shown in **Figure 2**.

In order to compute the BED in case of non-constant dose administration the following formula was applied (Fowler, 2014):

(7) $$\mathrm{BED}=\sum_{i=1}^{n}\left(1+\frac{d}{\alpha /\beta }\right)$$

where *i* is the index that scans the n irradiation composing the overall treatment. All the simulations were performed considering a relative tumor volume evolution, in other words the initial tumor volume was set equal to 100% at *t* = 0 (first irradiation).

## Results

**FIGURE 3:**
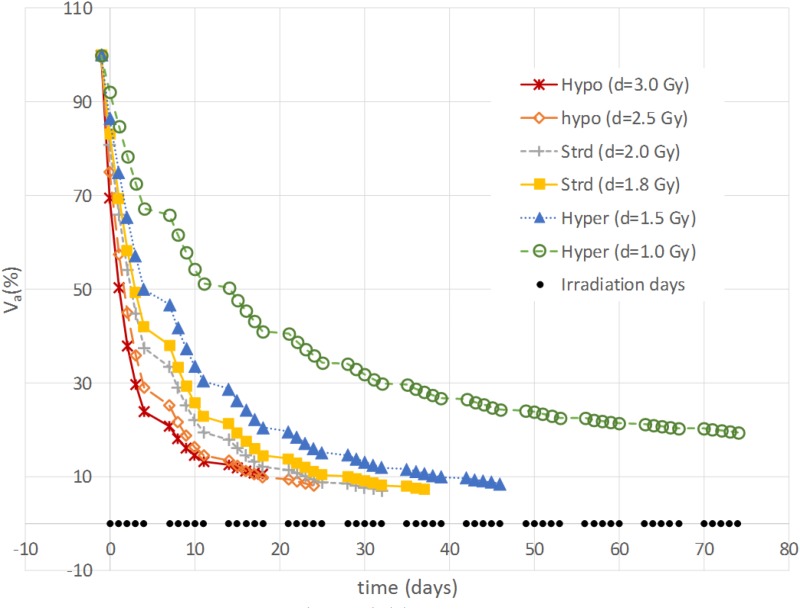
Example of active volume evolution. Viable tumor regression according to different constant-dose fractionation schemes for Patient B.

The aim of the treatment is to necrotize the tumor, therefore, the overall final mass may be not as relevant as the residual active portion since, eventually, the necrotic volume is going to be washed out physiologically. As a matter of fact, an advantage of using the proposed model is to be able to tell the two components apart and to focus the analysis on the viable volume only. Since the model was trained on the overall tumor volume only, we started investigating whether analyzing the active volume instead of the total volume implied the selection of a different optimal treatment according to the criterion of the maximum final volume reduction. The two criteria (total volume vs. active volume reduction) resulted in agreement in all the cases except for patient B (**Table 1**) where according to *V_t_* the best treatment was *d* = 1.8 Gy, while, considering *V_a_*, *d* = 2 Gy was to be selected. It has to be remarked that for patient B the two treatments (*d* = 1.8 Gy and *d* = 2 Gy) provided comparable performances (final volume variation ≅ 0.5) as shown in **Figure 3**, and that both are considered standard regimens (strd) in clinical practice. Given the abovementioned considerations, the result presentation and the following discussion will focus on the active volume only. Among the seven patients, three benefited more from hypofractionated treatment, two showed a larger regression by means of the standard schedule and the remaining two performed better under hypofractionated regimen (see **Table 2**).

**Table 2:** The final volume tumor volume is shown for the constant-dose treatments according to the patient and the dose per fraction administered (1 Gy < *d* < 3 Gy) considering the overall tumor [*V_t_*(t*_e_*), upper panel] and the active portion [*V_a_*(*t_e_*), lower panel], respectively.

Patient	*d* = 1	*d* = 1.5	*d* = 1.8	*d* = 2	*d* = 2.5	*d* = 3	*c[(d,V_t_(t_e_)]*	Best treatment	

Final overall volume – *V_t_*(*t_e_*) [%]									
A	73.65	48.22	40.27	36.89	32.22	**31.04**	−0.842	*d* = 3	hypo
B	20.95	10.80	**9.67**	10.15	12.27	18.01	−0.495	*d* = 1.8	strd
C	45.12	29.37	26.53	**26.24**	27.71	31.35	−0.653	*d* = 2	strd
D	**1.00**	1.66	2.42	3.10	5.18	8.32	0.984	*d* = 1	hyper
E	**5.33**	9.19	12.67	15.66	22.93	31.71	0.990	*d* = 1	hyper
F	51.50	28.15	22.83	**22.44**	23.30	30.36	−0.696	*d* = 2.5	hypo
G	63.68	47.39	43.16	**42.36**	42.85	47.61	−0.725	*d* = 2.5	hypo

**Patient**	***d* = 1**	***d* = 1.5**	***d* = 1.8**	***d* = 2**	***d* = 2.5**	***d* = 3**	***c[d,V_a_(t_e_)]***	**Best treatment**	

**Final active volume** – *V_a_*(*t_e_*) [%]									

A	73.28	47.84	39.96	36.49	31.80	**30.44**	−0.883	*d* = 3	hypo
B	19.54	8.56	7.34	**7.07**	8.18	10.48	−0.515	*d* = 2	strd
C	44.52	28.09	25.29	**24.54**	26.00	29.41	−0.571	*d* = 2	strd
D	**0.88**	1.48	2.24	2.87	4.87	7.17	0.974	*d* = 1	hyper
E	**4.12**	7.21	10.58	13.07	20.06	26.84	0.989	*d* = 1	hyper
F	49.44	22.32	16.34	13.72	**12.18**	13.07	−0.794	*d* = 2.5	hypo
G	62.82	45.31	40.64	38.89	**38.38**	40.44	−0.754	*d* = 2.5	hypo

*Only three patients (B, D, and E) reached the 80% reduction (final volume <20%), in bold the lowest final volume. The last two columns of both tables show the correlation between the dose per fraction and the tumor at the end of irradiation (t_e_) and the best treatment according to the lowest final volume criterion. Considering either the active or total volume the optimal treatment changes only for patient B as underlined.*

**Table 3:** BED of the partial treatment.

BED (Gy)						
**Patient**	***d* = 1**	***d* = 1.5**	***d* = 1.8**	***d* = 2**	***d* = 2.5**	***d* = 3**
A	–	–	–	–	–	–
B	58.3	27.6	23.4	24.0	25.0	27.3
C	–	–	–	–	–	–
D	11.0	10.3	12.7	9.6	12.5	11.7
E	24.2	25.9	29.7	31.2	–	–
F	–	–	50.9	48.0	43.7	42.9
G	–	–	–	–	–	–

*For each of the four patients able to reach the 80% tumor reduction (B, D, E, and F) the BED is computed considering an early treatment stop as soon as the V_a_(t_e_) < 20 threshold is reached.*

**FIGURE 4:**
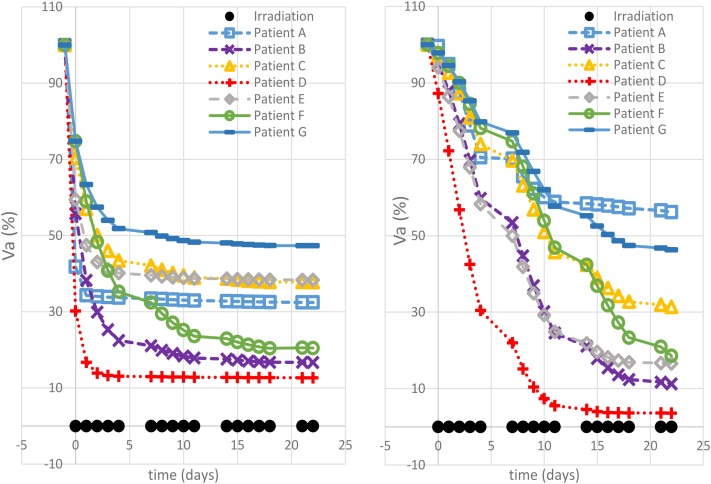
Non-constant dose administration – simulation results. The viable tumor regression is shown for each patient simulating a decreasing dose administration trend (left panel) and an increasing one (right panel).

Different correlation values [*c*(*d,V*)] between dose delivered and final volume size were found, which reflected the value of the growth rate. The hypo group [patients A, F, and G, *c*(*d,V_a_*(*t_e_*)) < −0.75] featured ρ ≥ 0.1, for the strd group [patients B and C, *c*(*d,V_a_*(*t_e_*)) ≅ −0.5] 0.07 < ρ < 0.08 and for the hyper group ρ < 0.05 [patients D and E, *c*(*d,V_a_*(*t_e_*)) > 0.97]. In other words, the growth rate (ρ) appeared to be a discriminating factor in the treatment selection. Four patients (B, D, E, and F) reached 80% active volume reduction [*V_a_*(*t_e_*) < 20] at least with one fractionation scheme. It has to be pointed out that all of them obtained such reduction with the standard treatment even if in some cases other fractionation schemes allowed even a larger regression. Therefore, in case of *V_a_*(*t_e_*) < 20, we introduced a secondary treatment ranking tacking into consideration an early radiotherapy interruption once the 80% reduction is achieved, checking the BED delivered until then. This approach can be used in case the main goal of EBRT is to reach a suitable tumor reduction before administering other treatments (e.g., brachytherapy, chemotherapy, etc.). It allows a reduction in the dose administration and, reasonably, a decrease in the NTCP including, for instance, toxicity and acute reactions. The results of such analysis are shown in **Table 3**, where the new BED is provided and the best treatment option, according to the BED minimizing criterion, is highlighted. The results of the non-constant dose administration are depicted in **Figure 4**, where the simulation of the active tumor evolution is shown for each patient according to the decreasing (upper panel) and increasing (lower panel) trends, respectively. Neither of the treatment did systematically outperform the other, as in the constant-dose fractionation the optimal strategy was to be selected individually for each patient. Despite the fact that both fractionation involved exactly the same dose administration (in the reverse order) the final volumes of the same patient varied on average of about 10% considering the initial volume $\left(\left|{\overset{\wedge }{V}}_{a}(t_{e})-{\overset{\vee }{V}}_{a}(t_{e})\right|\right)$ and over 40% with respect to the final volume itself $\left(\frac{\left|{\overset{\wedge }{V}}_{a}(t_{e})-{\overset{\vee }{V}}_{a}(t_{e})\right|}{\left({\overset{\wedge }{V}}_{a}(t_{e})-{\overset{\vee }{V}}_{a}(t_{e})\right)/2}\right)$, where ${\overset{\wedge }{V}}_{a}$(*t_e_*) and ${\overset{\vee }{V}}_{a}$(*t_e_*) are the final active volumes for the increasing and decreasing treatment, respectively. The treatment featured 0.5 Gy < *d* < 4.5 Gy and a Δ*d* = 0.25 between consecutive fractions.

### Major Findings

The simulations performed in this study, using a model trained on clinical patient data, highlight the potential of exploiting mathematical modeling in radiotherapy planning. It is possible to identify four main findings: (1) each virtual patient reacts differently when administered with the same hypo/hyper fractionated regimen; (2) the maximum tumor reduction was obtained with different fractionation schemes for each of the seven patients; (3) an early treatment stop for some patients could theoretically allow a reduction of BED administration, while ensuring a greater reduction (80%) of the tumor volume; (4) reversal of the dose administration order in a non-constant dose schedule, causes an average variation of 40% in the final active volume.

First of all, we noticed that the same treatment can behave very differently depending on the patient characteristics, even if it was expected, given that the data used for the training itself showed large variability even in the final regression (20–90%) (Belfatto et al., 2017). For example, the hyperfractionation (*d* = 1) leads to a reduction of less than 30% for patient A and more than 99% for patient D (see **Table 2**). Observing the model parameters, it can be easily seen that these patients are those with the lowest (patient D) and the largest growth rate (patient A), respectively. Therefore, it is perfectly reasonable that a longer treatment period (hyper-fractionation) would result in a greater tumor progression in A than D reducing the effectiveness of irradiation in the second.

The opposite analysis, which studies the best treatment option for each patient, is probably the most interesting because of its clear potential impact in clinical practice: the personalization of treatment. The study showed that the same patient administered with the same BED by a different fractionation regime could result in a variation of more than 40% in the reduction of the final volume, e.g., patient A: final active volume equal to 73% if *d* = 1 and 30% if *d* = 3 (see **Table 2**). We hypothesized, for the purpose of this analysis, that the best treatment option for each patient was the one that provides the widest regression (Lee et al., 2014). The tumor growth rate plays a key role, allowing discrimination among patients who could benefit most from hypo-, hyper- or standard fractionation, according to the literature (Fowler, 2001). A previous study, which investigated the possibility of selecting altered regimens (hypo/hyper fractionation) based on patient-specific models, suggested that the carrying capacity was the most relevant factor (Prokopiou et al., 2015). The authors introduced a proliferation saturation index (PSI) that describes the tumor volume compared to the carrying capacity, assuming the higher the ratio the lower the proliferation of cells, since the tumor is close to its saturation. PSI showed high inverse correlation with the radiotherapy response, in agreement with the fact that low proliferation implies reduced radiosensitivity and also seemed to identify the optimal radiation regimen. In our case, patients A and D had a PSI of 0.45 and 0.33, respectively. Those were actually the smallest PSI values in the dataset, however, the two patients showed opposite behavior as described above. This apparent inconsistency can have more explanations. The curve used in Prokopiou et al. (2015) to fit the data was a Logistic function instead of a Gompertzian as in the present study, so even if the parameters represent the same biological characteristic they have different values. The authors tested the model on a different cancer type (non-small cell lung cancer). They did not include the dynamics of dead-cell clearance and considered a constant carrying capacity. In particular, they acknowledged that the constant carrying capacity is a limitation of their work, which we have overcome in the present study (Prokopiou et al., 2015). The selection of the optimal fractionation scheme could also be performed using different criteria such as the minimum BED to be administered to obtain a predefined regression (80%). This approach can be used only for the treatments that actually reach the selected threshold of tumor reduction. The analysis showed that in some cases the BED could be limited to 1/6 of its standard value (60 Gy) considering a 1.8 Gy × 28-fractions treatment, implying a reduced irradiation of the healthy tissue surrounding the gross tumor volume lowering the NTCP. The minimum-BED criterion can be exploited especially in case of multi-modality treatments, when the EBRT objective is not to completely sterilize the tumor but to reduce it sufficiently to proceed with the following treatment step (e.g., either brachytherapy or surgery). It is important to note that this would also reduce the treatment cost per patient, which is a critical aspect in modern medicine, while ensuring a greater tumor regression.

In conclusion, according to the model, not only the amount of dose per fraction but also the order used to administer it is relevant to the endpoint (final active volume). Patients A (largest ρ) and patient D (lowest ρ) still exhibit opposite behaviors. Faster growth requires a very aggressive treatment in the initial stages to limit its effect while, in the case of slower growth, higher doses are required when the vasculature impairments has reduced the tumor oxygenation and, consequently, its radiosensitivity.

### Limitations

This work presents some limitations that can be summarized as: (1) reduced number of virtual patients; (2) model training limited to the overall tumor volume only and no separate validation for the viable portion; (3) model training based on a standard fractionation regimen only (1.8 Gy × 28 fractions); and (4) lack of an explicit definition of the irradiation effects on healthy tissues.

The number of virtual patients was limited by the number of actual patients on whom the model had been trained in the previous study. We are aware that the reduced number of parameter sets does not allow meaningful statistics. However, the point of this study was to highlight the need for personalization in radiotherapy fractionated regimens and propose a mathematical framework to support the best regimen selection. The wide variability in the results of simulations of the same treatment on different patients, as well as of the same patient administered with different treatments, supports our initial hypothesis and makes the results of this work promising while preliminary. Future studies increasing the patient cohort are in order to confirm them.

Although model training was performed minimizing the error between the measured volume and the sum of predicted active and necrotic volumes, the validation was performed by means of oxygen-related indexes derived from the 3D-Doppler images (Huang et al., 2010). A high correlation was found between the model prediction of tumor oxygenation, which is the product of a complex network of mutual interconnections also on the active tumor and of the indices mentioned above in some patients. Therefore, the validation of predicted oxygenation is an indirect proof of the accurate estimate of the viable portion dynamics. In this work, we have also showed that the selection of the best treatment, according to the maximum regression criterion, would not have differed considering the total volume of the tumor instead of the viable portion only.

The generalization of a model trained on a specific dose schedule to other fractionation schemes is not straightforward. We are aware that some radiobiological parameters may be dependent on the dose (Williams et al., 1985; Nahum et al., 2003; Astrahan, 2008) and that this can limit the prediction reliability. However, we have addressed this issue by working within a reasonable range of dose administration (0.5 Gy ≤*d* ≤ 4.5 Gy). The fact that the treatment is administered in a one-size-fits-all solution, which is exactly the problem addressed in this work, has prevented the observation of the effects due to different regimens. In order to include them, a multi-center repository should be used.

The toxicity of healthy tissue is a critical aspect of radiotherapy that must be taken into account. In order to provide a realistic scenario for each of the presented simulations, the corresponding dose profile should have been generated, the dose delivered to the organs at risk assessed and its effects modeled. A correlation between this and the long terms effects would finally have allowed a new treatment selection criterion. This assessment was beyond the scope of our analysis since it largely depends on the irradiation modalities (e.g., number of beams, use of IMRT, etc.). Therefore, we focused only on providing realistic treatments, forcing the BED to be ≅60 in addition to the limitation of the maximum dose per fraction.

### Final Remarks

**FIGURE 5:**
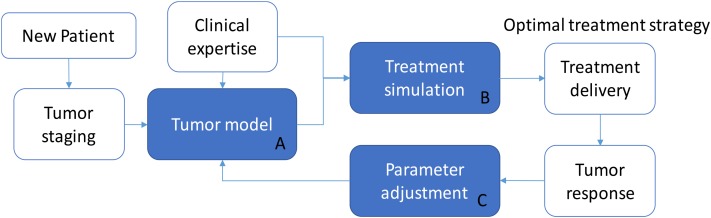
Model supported treatment planning.

Although this is a simulated study, the proposed model was trained on real patient data. In principle, this approach makes it possible to predict the effects of any irradiation treatment on a generic patient, provided that the corresponding parameter signature (ρ, *T*_1/2_, $\hat{k}$, and γ) is identified. We hypothesize that a model-supported treatment planning tool can be built on the basis of the model presented (**Figure 5**) by providing a personalized dose delivery regimen. In this scenario, the real treatment can be updated thanks to the monitoring of tumor response, which can guide the adjustment of model parameters, and this in turn can determine a change in the treatment strategy at run-time. In detail, while a new patient enters the protocol, tumor staging and patient profile (e.g., age, pre-existing conditions, etc.) may determine the selection of an appropriate group-specific model (block A) defined by a specific set of parameters, which represent a consistent patient population (tumor growth rate, radio-sensitivity, carrying capacity). The support to select a tailored treatment is ensured thanks to the simulation in bundle of different irradiation protocols (block B) which can feature different dose profiles and inter-fraction interruptions as well. They can be ranked according to either the maximum regression by selecting the treatment leading to *min*(*V_a_*(*t_e_*)) or according to the minimum BED criterion [*min*(*BED*(*t_e_*))], depending on the specific treatment requirements. For example, if the aim is to achieve a certain tumor shrinkage in order to allow surgery, the fractionation scheme leading to an adequate reduction while minimizing the dose delivered to the patient (according to the model predictions), should be promoted. After selecting the optimal treatment regimen, in accordance with clinical expertize and institutional guidelines, the patient begins the curative path. During the radiation course, the tumor evolution is monitored by means of the acquisition of morphologic/functional images. The sensible data, extracted from images, can be used to better tune the parameters of the group-specific model to cope with the patient-specific response (block C). An example of model refinements by means of a parameter adaptation approach along the treatment administration is described in Belfatto et al. (2016c). New simulations can be then performed and the treatment strategy may be revised accordingly (back to block A, with updated parameters). In principle, this approach could exploit the potential of tumor modeling to allow the treatment to be personalized more objectively and quantitatively than the standard profile. Moreover, it can take advantage of data already acquired for tumor monitoring and patient positioning. This makes the model-based treatment support system interesting and worthy of further investigation, mandatory for its translation into clinics.

## Author Contributions

AB developed the models, processed the data, and contributed to results analysis and paper writing. BJ-F provided the raw data, contributed to data preprocessing, and revised the paper from a clinical point of view. GB contributed to physiologic and clinical contents and revised the paper from a technical point of view. PC contributed to the model developments, results analysis, and paper writing.

## Conflict of Interest Statement

The authors declare that the research was conducted in the absence of any commercial or financial relationships that could be construed as a potential conflict of interest.

## References

[B1] Ahmed K. A. Correa C. R. Dilling T. J. Rao N. G. Shridhar R. Trotti A. M. (2014). Altered fractionation schedules in radiation treatment: a review. *Semin. Oncol.* 41 730–750. 10.1053/j.seminoncol.2014.09.012 25499633

[B2] Astrahan M. (2008). Some implications of linear-quadratic-linear radiation dose-response with regard to hypofractionation. *Med. Phys.* 35 4161–4172. 10.1118/1.2969065 18841869

[B3] Belfatto A. Riboldi M. Ciardo D. Cattani F. Cecconi A. Lazzari R. (2016a). Kinetic models for predicting cervical cancer response to radiation therapy on individual basis using tumor regression measured in vivo with volumetric imaging. *Technol. Cancer Res. Treat.* 15 146–158. 10.1177/1533034615573796 25759423

[B4] Belfatto A. Riboldi M. Ciardo D. Cattani F. Cecconi A. Lazzari R. (2016b). Modeling the interplay between tumor volume regression and oxygenation in uterine cervical cancer during radiotherapy treatment. *IEEE J. Biomed. Health Inform.* 20 596–605. 10.1109/JBHI.2015.2398512 25647734

[B5] Belfatto A. Riboldi M. Ciardo D. Cecconi A. Lazzari R. Jereczek-Fossa B. A. (2016c). Adaptive mathematical model of tumor response to radiotherapy based on CBCT data. *IEEE J. Biomed. Health Inform.* 20 802–809. 10.1109/JBHI.2015.2453437 26173223

[B6] Belfatto A. Vidal Urbinati A. M. Ciardo D. Franchi D. Cattani F. Lazzari R. (2017). Comparison between model-predicted tumor oxygenation dynamics and vascular-/flow-related Doppler indices. *Med. Phys.* 44 2011–2019. 10.1002/mp.12192 28273332

[B7] Belfatto A. White D. A. Zhang Z. Zhang Z. Cerveri P. Baroni G. (2015). “Mathematical modeling of tumor response to radiation: radio-sensitivity correlation with BOLD, TOLD, ΔR 1 and ΔR 2^∗^ investigated in large Dunning R3327-AT1 rat prostate tumors. In engineering in Medicine and Biology Society (EMBC),” in *Proceedings of the 37th Annual International Conference of the IEEE*, Milano, 3266–3269. 10.1109/EMBC.2015.7319089 26736989

[B8] Bellomo N. Bellouquid A. Delitala M. (2004). Mathematical topics on the modelling complex multicellular systems and tumor immune cells competition. *Math. Mod. Methods Appl. Sci.* 14 1683–1733. 10.1142/S0218202504003799

[B9] Boondirek A. Triampo W. Nuttavut N. (2010). A review of cellular automata models of tumor growth. *Int. Math. Forum* 5 3023–3029.

[B10] Burnet N. G. Thomas S. J. Burton K. E. Jefferies S. J. (2004). Defining the tumour and target volumes for radiotherapy. *Cancer Imag.* 4 153–161. 10.1102/1470-7330.2004.0054 18250025 PMC1434601

[B11] Chow E. Harris K. Fan G. Tsao M. Sze W. M. (2007). Palliative radiotherapy trials for bone metastases: a systematic review. *J. Clin. Oncol.* 25 1423–1436. 10.1200/JCO.2006.09.5281 17416863

[B12] Colombo N. Carinelli S. Colombo A. Marini C. Rollo D. Sessa C. (2012). Cervical cancer: ESMO clinical practice guidelines for diagnosis, treatment and follow-up. *Ann. Oncol.* 23(Suppl. 7), vii27–vii32. 10.1093/annonc/mds268 22997451

[B13] Contin P. Kulu Y. Bruckner T. Sturm M. Welsch T. Müller-Stich B. P. (2014). Comparative analysis of late functional outcome following preoperative radiation therapy or chemoradiotherapy and surgery or surgery alone in rectal cancer. *Int. J. Colorectal. Dis.* 29 165–175. 10.1007/s00384-013-1780-z 24136155

[B14] Cornelis F. Saut O. Cumsille P. Lombardi D. Iollo A. Palussiere J. (2013). In vivo mathematical modeling of tumor growth from imaging data: soon to come in the future? *Diagn. Interv. Imag.* 94 593–600. 10.1016/j.diii.2013.03.001 23582413

[B15] Dale R. G. (1996). Dose-rate effects in targeted radiotherapy. *Phys. Med. Biol.* 41 1871–1884. 10.1088/0031-9155/41/10/001 8912367

[B16] Deschner E. E. Gray L. H. (1959). Influence of oxygen tension on x-ray-induced chromosomal damage in Ehrlich ascites tumor cells irradiated in vitro and in vivo. *Radiat. Res.* 11 115–146. 10.2307/3570739 13668067

[B17] Fowler J. F. (1989). The linear-quadratic formula and progress in fractionated radiotherapy. *Br. J. Radiol.* 62 679–694. 10.1259/0007-1285-62-740-679 2670032

[B18] Fowler J. F. (2001). Biological factors influencing optimum fractionation in radiation therapy. *Acta Oncol.* 40 712–717. 10.1080/02841860152619124 11765065

[B19] Fowler J. F. (2014). 21 years of biologically effective dose. *Br. J. Radiol.* 83:991. 10.1259/bjr/31372149 PMC3473681 20603408

[B20] Fyles A. W. Milosevic M. Wong R. Kavanagh M. C. Pintilie M. Sun A. (1998). Oxygenation predicts radiation response and survival in patients with cervix cancer. *Radiother. Oncol.* 48 149–156. 10.1016/S0167-8140(98)00044-9 9783886

[B21] Ghaffari P. Mardinoglu A. Nielsen J. (2015). Cancer metabolism: a modeling perspective. *Front. Physiol.* 6:382 10.3389/fphys.2015.00382 PMC4679931 26733270

[B22] Goldstein M. Kastan M. B. (2015). The DNA damage response: implications for tumor responses to radiation and chemotherapy. *Annu. Rev. Med.* 66 129–143. 10.1146/annurev-med-081313-121208 25423595

[B23] Gompertz B. (1825). On the nature of the function expressive of the law of human mortality, and on a new mode of determining the value of life contingencies. *Philos. Transac. R. Soc. Lond.* 115 513–583. 10.1098/rstl.1825.0026 25750242 PMC4360127

[B24] Haddad R. O’Neill A. Rabinowits G. Tishler R. Khuri F. Adkins D. (2013). Induction chemotherapy followed by concurrent chemoradiotherapy (sequential chemoradiotherapy) versus concurrent chemoradiotherapy alone in locally advanced head and neck cancer (PARADIGM): a randomised phase 3 trial. *Lancet Oncol.* 14 257–264. 10.1016/S1470-2045(13)70011-1 23414589

[B25] Heijkoop S. T. Langerak T. R. Quint S. Bondar L. Mens J. W. M. Heijmen B. J. (2014). Clinical implementation of an online adaptive plan-of-the-day protocol for nonrigid motion management in locally advanced cervical cancer IMRT. *Int. J. Rad. Oncol. Biol. Phys.* 90 673–679. 10.1016/j.ijrobp.2014.06.046 25151538

[B26] Hogea C. Davatzikos C. Biros G. (2008). An image-driven parameter estimation problem for a reaction–diffusion glioma growth model with mass effects. *J. Math. Biol.* 56 793–825. 10.1007/s00285-007-0139-x 18026731 PMC2871396

[B27] Huang Z. Mayr N. A. Yuh W. T. Lo S. S. Montebello J. F. Grecula J. C. (2010). Predicting outcomes in cervical cancer: a kinetic model of tumor regression during radiation therapy. *Cancer Res.* 70 463–470. 10.1158/0008-5472.CAN-09-2501 20068180 PMC2822442

[B28] Jones B. L. Westerly D. Miften M. (2015). Calculating tumor trajectory and dose-of-the-day using cone-beam CT projections. *Med. Phys.* 42 694–702. 10.1118/1.4905107 25652483

[B29] Karlsson M. (2004). The relationship between temporal variation of hypoxia, polarographic measurements and predictions of tumour response to radiation. *Phys. Med. Biol.* 49 4463–4475. 10.1088/0031-9155/49/19/002 15552411

[B30] Keall P. J. Mageras G. S. Balter J. M. Emery R. S. Forster K. M. Jiang S. B. (2006). The management of respiratory motion in radiation oncology report of AAPM Task Group 76. *Med. Phys.* 33 3874–3900. 10.1118/1.2349696 17089851

[B31] Lee H. Ahn Y. C. Oh D. Nam H. Kim Y. I. Park S. Y. (2014). Tumor volume reduction rate measured during adaptive definitive radiation therapy as a potential prognosticator of locoregional control in patients with oropharyngeal cancer. *Head Neck* 36 499–504. 10.1002/hed.23328 23780633

[B32] Lyman J. T. (1985). Complication probability as assessed from dose-volume histograms. *Radiat. Res.* 104 S13–S19. 10.2307/3576626 3867079

[B33] McGale P. Taylor C. Correa C. Cutter D. Duane F. Ewertz M. (2014). Effect of radiotherapy after mastectomy and axillary surgery on 10-year recurrence and 20-year breast cancer mortality: meta-analysis of individual patient data for 8135 women in 22 randomised trials. *Lancet* 383 2127–2135. 10.1016/S0140-6736(14)60488-8 24656685 PMC5015598

[B34] Nahum A. E. Movsas B. Horwitz E. M. Stobbe C. C. Chapman J. D. (2003). Incorporating clinical measurements of hypoxia into tumor local control modeling of prostate cancer: implications for the α/β ratio. *Int. J. Radiat. Oncol. Biol. Phys.* 57 391–401. 10.1016/S0360-3016(03)00534-0 12957250

[B35] Oberhardt M. A. Gianchandani E. P. (2015). Genome-scale modeling and human disease: an overview. *Front. Physiol.* 5:527 10.3389/fphys.2014.00527 PMC4304257 25667572

[B36] Paganelli C. Summers P. Bellomi M. Baroni G. Riboldi M. (2015). Liver 4DMRI: a retrospective image-based sorting method. *Med. Phys.* 42 4814–4821. 10.1118/1.4927252 26233208

[B37] Prokopiou S. Moros E. G. Poleszczuk J. Caudell J. Torres-Roca J. F. Latifi K. (2015). A proliferation saturation index to predict radiation response and personalize radiotherapy fractionation. *Radiat. Oncol.* 10:159. 10.1186/s13014-015-0465-x 26227259 PMC4521490

[B38] Schaerer J. Fassi A. Riboldi M. Cerveri P. Baroni G. Sarrut D. (2012). Multi-dimensional respiratory motion tracking from markerless optical surface imaging based on deformable mesh registration. *Phys. Med. Biol.* 57 357–373. 10.1088/0031-9155/57/2/357 22170786

[B39] Seregni M. Cerveri P. Riboldi M. Pella A. Baroni G. (2012). Robustness of external/internal correlation models for real-time tumor tracking to breathing motion variations. *Phys. Med. Biol.* 57 7053–7074. 10.1088/0031-9155/57/21/7053 23053391

[B40] Sharfo A. W. M. Breedveld S. Voet P. W. Heijkoop S. T. Mens J. W. M. Hoogeman M. S. (2016). Validation of fully automated VMAT plan generation for library-based plan-of-the-day cervical cancer radiotherapy. *PLoS One* 11:e0169202. 10.1371/journal.pone.0169202 28033342 PMC5199117

[B41] Soerjomataram I. Ervik M. Dikshit R. Eser S. Mathers C. Rebelo M. (2012). “GLOBOCAN 2012 v1. 0, Cancer incidence and mortality worldwide: IARC CancerBase No. 11,” in *Proceedings of the International Agency for Research on Cancer. World Health Organisation*, Geneva.

[B42] Stamatakos G. S. Dionysiou D. D. (2009). Introduction of hypermatrix and operator notation into a discrete mathematics simulation model of malignant tumour response to therapeutic schemes in vivo. Some operator properties. *Cancer Inform.* 7 239–251. 10.4137/CIN.S2712 20011462 PMC2791491

[B43] Stocks T. Hillen T. Gong J. Burger M. (2014). A stochastic model for the normal tissue complication probability (NTCP) in radiation treatment of cancer. *arXiv.* 27591250 10.1093/imammb/dqw013

[B44] Thames H. D. Peters L. T. Withers H. R. Fletcher G. H. (1983). Accelerated fractionation vs hyperfractionation: rationales for several treatments per day. *Int. J. Radiat. Oncol. Biol. Phys.* 9 127–138. 10.1016/0360-3016(83)90089-5 6833014

[B45] Williams M. V. Denekamp J. Fowler J. F. (1985). A review of αβ ratios for experimental tumors: implications for clinical studies of altered fractionation. *Int. J. Radiat. Oncol. Biol. Phys.* 11 87–96. 10.1016/0360-3016(85)90366-9 3881377

